# Cannabis use and mood disorders: a systematic review

**DOI:** 10.3389/fpubh.2024.1346207

**Published:** 2024-04-09

**Authors:** Maryam Sorkhou, Eliza L. Dent, Tony P. George

**Affiliations:** ^1^Institute for Mental Health Policy and Research at CAMH, Toronto, ON, Canada; ^2^Department of Psychiatry, Institute of Medical Sciences, University of Toronto, Toronto, ON, Canada; ^3^Department of Psychology, McGill University, Montreal, QC, Canada

**Keywords:** cannabis, major depressive disorder, bipolar disorder, depression, mania, suicidality

## Abstract

**Background:**

Problematic cannabis use is highly prevalent among people with mood disorders. This underscores the need to understand the effects of cannabis and cannabinoids in this population, especially considering legalization of recreational cannabis use.

**Objectives:**

We aimed to (1) systematically evaluate cross-sectional and longitudinal studies investigating the interplay between cannabis use, cannabis use disorder (CUD), and the occurrence of mood disorders and symptoms, with a focus on major depressive disorder (MDD) and bipolar disorder (BD) and; (2) examine the effects of cannabis on the prognosis and treatment outcomes of MDD and BD.

**Methods:**

Following PRISMA guidelines, we conducted an extensive search for English-language studies investigating the potential impact of cannabis on the development and prognosis of mood disorders published from inception through November 2023, using EMBASE, PsycINFO, PubMed, and MEDLINE databases.

**Results:**

Our literature search identified 3,262 studies, with 78 meeting inclusion criteria. We found that cannabis use is associated with increased depressive and manic symptoms in the general population in addition to an elevated likelihood of developing MDD and BD. Furthermore, we observed that cannabis use is linked to an unfavorable prognosis in both MDD or BD.

**Discussion:**

Our findings suggest that cannabis use may negatively influence the development, course, and prognosis of MDD and BD. Future well-designed studies, considering type, amount, and frequency of cannabis use while addressing confounding factors, are imperative for a comprehensive understanding of this relationship.

**Systematic review registration:**

https://www.crd.york.ac.uk/prospero/display_record.php?ID=CRD42023481634.

## Introduction

Mood disorders represent a substantial global mental health challenge, with major depressive disorder (MDD) affecting up to 15% of the population, or approximately 300 million individuals worldwide (1). Moreover, bipolar disorder (BD) exhibits a lifetime prevalence of 1.9%, with an additional 4.6% of individuals experiencing subclinical presentations (e.g., cyclothymia) (2). These disorders typically start in adolescence and follow a chronic course, commonly leading to substantial functional impairment (3, 4). Moreover, both MDD and BD contribute significantly to years marked by disability, underscoring their profound impact on quality of life and well-being.

Concurrently, the landscape of cannabis use is evolving globally, with 192 million people reporting its use in 2018 (5), and rates of cannabis use disorder (CUD) estimated at ~3% (6). Notably, the rates of cannabis use among people with mood disorders are increasing at a faster rate compared to those without such conditions (7, 8). For instance, between 2005 and 2006, people with MDD showed a 30% higher likelihood of daily cannabis use than their non-depressed counterparts. However, this proportion surged to 216% between 2015 and 2016 (9).

This evolving trend in cannabis use among people with mood disorders is particularly relevant because it raises pivotal questions on how the compounds in cannabis may influence the development, course, and treatment of mood disorders. Cannabis contains ~400 compounds, with over 100 identified cannabinoids (10). Two extensively studied compounds in cannabis are delta-9-tetrahydrocannabinol (THC) and cannabidiol (CBD). THC is known for producing psychotomimetic and anxiogenic effects (11, 12), whereas CBD is believed to possess antipsychotic, pro-cognitive, and anti-anxiety properties (13, 14). These compounds target the endocannabinoid system, which is involved in brain development, cognition, and emotion regulation (14, 15). Manipulating this system through cannabis or exogenous cannabinoids may affect the pathophysiology and progression of mental health disorders.

Concerning the effects of cannabis use on mood disorders, a complex relationship exists, characterized by proposals of both harmful and therapeutic effects in animal and human populations. Evidence indicates that a significant number of people report relief of depressive symptoms during acute cannabis intoxication. A recent meta-analysis found that approximately 34% of people using medical cannabis reported the alleviation of mood symptoms, potentially including attenuation of cannabis withdrawal, as their primary reason for using cannabis (16). However, long-term, or heavy cannabis use is also associated with the exacerbation of mood symptoms (17, 18). This paradox raises questions about how cannabis might impact the development and progression of mood disorders.

Evidence on cannabis use and mood disorders is limited and, at times, inconsistent. While some studies have not established an association between self-reported cannabis use and MDD or BD following confounder adjustments (19), other observational studies have identified a positive relationship between cannabis use and subsequent MDD, BD, and manic symptoms (20, 21). Therefore, the objectives of this systematic review are to: (1) comprehensively evaluate cross-sectional and longitudinal studies investigating the interplay between cannabis use, cannabis use disorder (CUD), and the occurrence of mood disorders and symptoms, with a focus on MDD and BD and; (2) examine the effects of cannabis, including, tetrahydrocannabinol (THC) and cannabidiol (CBD), on prognosis, treatment outcomes, and non-clinical aspects such as cognition and neural functioning in MDD and BD.

## Methods

### Search strategy

This systematic review was conducted according to Preferred Reporting Items for Systematic Reviews and Meta-Analyses (PRISMA) guidelines (22). Using EMBASE, PsycINFO, PubMed, and MEDLINE, we identified English-language studies published from inception through November 2023. A combination of free-text terms and medical subject heading terms were used for the subject search. Search terms (found in the title or abstract) included the following: ‘cannabis’, ‘tetrahydrocannabinol’, ‘cannabidiol’, ‘cannabinoid’, ‘bipolar disorder’, ‘major depressive disorder’, ‘mania’, and ‘suicide’. Furthermore, we manually searched through the reference lists of all eligible articles and relevant systematic reviews to retrieve additional studies.

Title and abstract screening in addition to full-text review were conducted by two of the reviewers (MS and ED). Any uncertainties were reviewed and reconciled by the senior author (TPG). This systematic review was registered on PROSPERO (ID: CRD42023481634).

### Inclusion and exclusion criteria

We considered observational studies focusing on the influence of cannabis on the initiation of mood disorders, giving priority to both longitudinal and cross-sectional studies. Included studies were required to assess major depressive disorder (MDD), bipolar disorder (BD), mania, hypomania, suicidality, dysthymia, or depressive symptoms using validated clinical tools. For studies investigating the development of mood disorders or symptoms, we additionally limited our scope to studies conducted in the general population. In the context of studies evaluating the effects of cannabinoids on the treatment outcomes and prognosis of MDD and BD, we considered both experimental and observational studies. Despite the anticipated limited availability of large, well-controlled randomized controlled trials (RCTs) in this domain, our search maintained inclusivity to comprehensively assess the available literature.

We excluded the following from our review: (a) literature reviews, meta-analyses, dissertations, commentaries, conference presentations, abstracts, and case studies; (b) studies examining the effect of mood disorders on subsequent cannabis use; (c) studies lacking a baseline measure of cannabis use; (d) animal models of cannabis use; (e) studies involving a special population (e.g., persons living with human immunodeficiency virus) and; (f) non-English publications.

### Data extraction and quality assessment

Data was extracted by one reviewer (MS). The following variables from each article were recorded: author, publication year, study design, number of participants, age range of participants, follow-up time, cannabis use measure, mood disorder/symptom measure, and relevant findings.

The Newcastle-Ottawa Scale (NOS) evaluated the quality of included observational studies (23). The NOS assesses non-randomized studies on a scale from 0–9 based on three main criteria: (1) selection of cases and controls (e.g., adequate definition, representativeness, and selection of cases); (2) comparability of cases and controls (e.g., adequate controlling or adjustment for confounding variables); and (3) ascertainment of exposure (e.g., adequate assurance that the cases were exposed to the variable of interest). Studies receiving a score ≥ 7 on the NOS are categorized as high quality, while studies receiving a score ≤ 4 are considered to exhibit low methodological quality (23).

We evaluated the quality of randomized controlled trials (RCTs) by assessing their risk of bias, through the Cochrane Collaboration Risk of Bias tool 2.0 (RoB) (24). For non-randomized interventions, the ROBINS-I tool was implemented (25). Each study was categorized as having high, low, or unclear risk in domains such as randomization, intervention adherence (deviation from intended interventions), missing outcome data, outcome measurement, and selective reporting. The worst grading on individual items defines the overall risk of bias for each single study.

## Results

### Study characteristics

**Figure 1:**
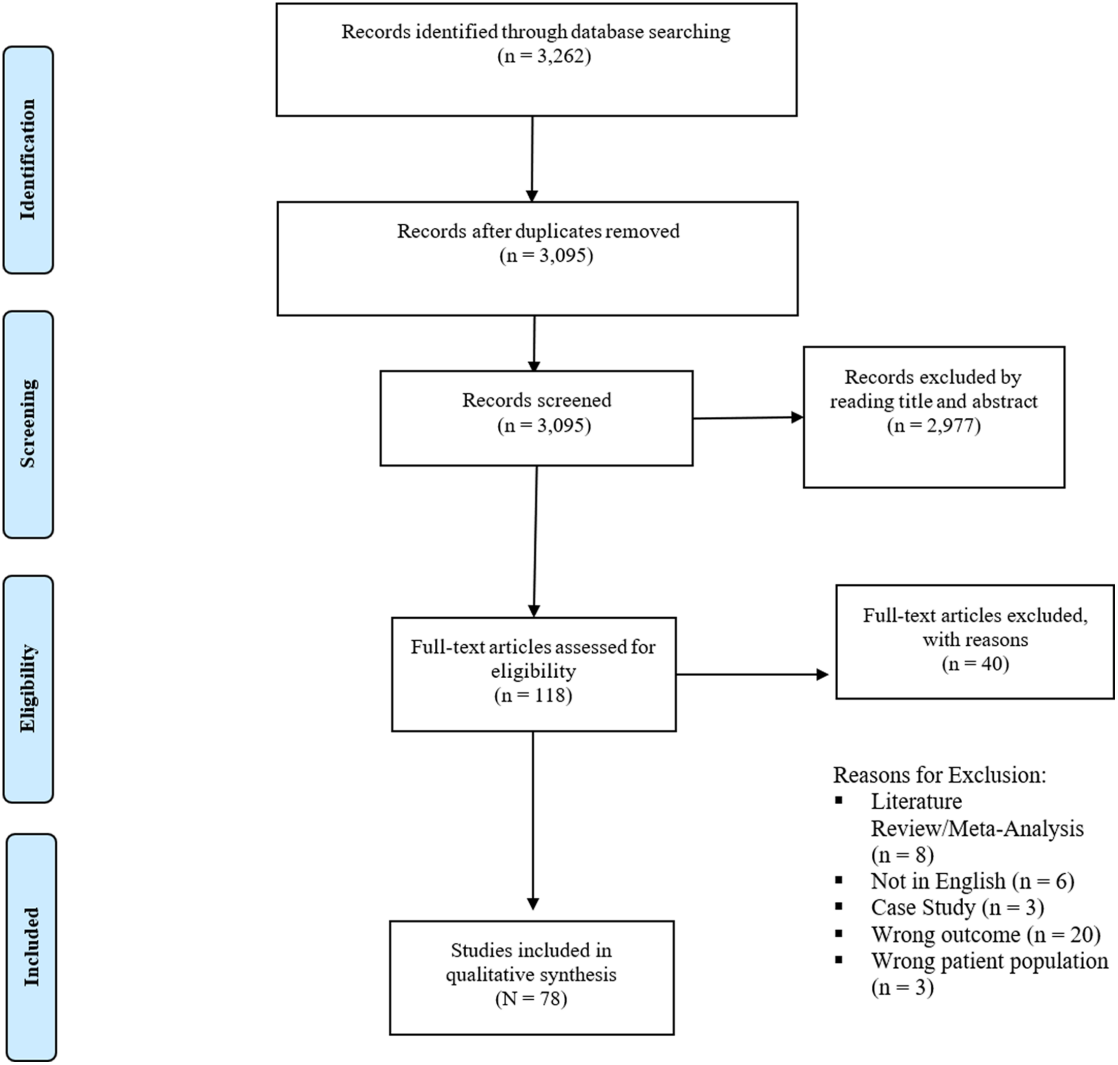
PRISMA flowchart of included studies.

Our initial literature search yielded a total of 3,262 studies. After eliminating 167 duplicates, we retained 3,095 records for screening based on their titles and abstracts. Following this screening process, 118 articles underwent full-text review. Of these, 78 papers met our inclusion criteria. Specific reasons for exclusion are listed in the PRISMA flowchart (Figure 1). The included studies spanned from 2001 to 2023 and were conducted in a wide range of countries. Among the 78 studies, there was one RCT, specifically examining the therapeutic potential of cannabidiol in treating BD (26). The remaining were observational or non-randomized in nature, with 52 adopting longitudinal designs and 25 utilizing cross-sectional designs. All but three publications (26–28) relied on self-report measures to assess cannabis use. Forty-eight studies examined the connection between cannabis use and MDD or depressive symptoms, 27 studies examined the relationship between cannabis use and BD or mania, and 3 studies examined the relationship between cannabis use and both depressive and manic symptoms. Table 1A outlines cross-sectional and longitudinal studies examining the association between cannabis use and MDD symptoms and diagnoses, while Table 1B addresses the corresponding relationship with manic symptoms and BD diagnoses. Table 2A provides a detailed account of cross-sectional and longitudinal studies that explore the impact of cannabis use on MDD while Table 2B examines the corresponding relationship within BD.

**Table 1A:** Summary of observational studies exploring the relationship between cannabis use, depressive symptoms, and MDD diagnoses (40 Studies, *N* = 1,015,726).

Author, year	Study population	Study design	Mood symptom or disorder measure	Cannabis use measure	Outcome	Relevant findings	NOS rating
Agrawal et al., 2017	*N* = 13,986 Australian Twins (aged 24–90)	Cross-sectional	DSM-IV	Self-report	MDD diagnosis, suicidal ideation	Among dizygotic and monozygotic twins, the twin who used cannabis frequently was more likely to report MDD and suicidal ideation compared to the twin who had used cannabis less frequently.	8
Bataineh et al., 2023	*N* = 1904 American adolescents (ages 13–18)	Longitudinal (5 year follow-up)	PHQ-9	Self-report	Depressive symptoms	Adolescents who reported any use of cannabis were significantly more likely to report depressive symptoms at younger ages compared to those who never used cannabis.	6
Bolanis et al., 2020	*N* = 1,606 Canadian adolescents (aged 15)	Longitudinal (5 year follow-up)	Mental Health and Social Inadaptation Assessment	Self-report	Depressive symptoms, suicidal ideation	Regular cannabis use at age 15 was associated with an increased risk of suicidal thoughts two years later. However, when accounting for the use of other substances, this relationship disappeared. Depression predicted weekly cannabis use, even after controlling for other substance use.	8
Carra et al., 2019	*N* = 527,446 American youth and adults (ages 12–64)	Repeated cross-sectional	DSM-IV	Self-report	Past-year major depressive episode	After controlling for time period, age, and gender, a dose dependent relationship between cannabis use and occurrence of a MDE emerged. Women were more likely than men to report concurrent past-year MDE and cannabis use.	7
Chabrol et al., 2020	*N* = 1,034 French postsecondary students (ages 18–30)	Cross-sectional	3-item suicide screen	CUDIT-R	Suicidal ideation	Cannabis use was not a significant predictor of suicidal ideation after controlling for confounds.	7
Crane et al., 2015	*N* = 1,263 American adolescents (ages 15–16)	Longitudinal (6 year follow-up)	CES-D	CUDIT-R	MDD symptoms	Baseline cannabis use was associated with depression symptoms at follow-up in males only.	7
Danielsson et al., 2016	*N* = 8,958 Swedish adults (ages 20–64)	Longitudinal (3 year follow-up)	DSM-IV, ICD-9	Self-report	MDD diagnosis	Adjusted for all confounders (alcohol and illicit drug use, education, family tension, place of upbringing, gender, and age) there was no relationship between baseline cannabis use and increased risk for MDD.	6
Davis et al., 2023	*N* = 2,234 American children (ages 12–13)	Longitudinal (13 annual waves)	Patient Health Questionnaire	Self-report	MDD Symptoms	Individuals who showed greater increases in cannabis use between two time points reported greater increases in depressive symptoms between subsequent time points.	7
Durdle et al., 2008	*N* = 226 MDMA users in America (mean age = 23.1)	Cross-sectional	DSM-IV	Drug Use Questionnaire, DSM-IV	MDD Diagnosis	Those with a diagnosis of MDD had higher rates of CUD than those without MDD.	6
Espada et al., 2011	*N* = 707 Spanish youth (Ages 11–16)	Cross-sectional	Child Depression Inventory	Substance Use Survey	Depressive Symptoms	Cannabis use was significantly associated with levels of depressive symptoms among youth.	7
Fairman et al., 2012	*N* = 173,775 American adults (ages 18 and older)	Cross-sectional	DSM-IV	Self-report	MDE	Early-onset (≥ age 18) and adult-onset cannabis smokers had in increased risk of a MDE to never cannabis smokers, even with covariate adjustment.	8
Feingold et al., 2015	*N* = 28,630 US adults (ages 18 and above)	Repeat cross-sectional	DSM-IV	Self-report	MDD incidence	Cannabis use was not significantly associated with increased incidence of MDD. Baseline MDD was associated with initiation of cannabis use.	
Fleming et al., 2008	*N* = 951 American adolescents (ages 12–14)	Longitudinal (4 annual waves)	Seattle Personality Questionnaire	Self-report	MDD symptoms	Increases in cannabis use was associated with increase in depressive symptom severity in boys but not among girls.	6
Frohe et al., 2019	*N* = 113,920 American adults (aged 18 and over)	Repeat cross-sectional (3 waves; 9 year-follow-up)	DSM-III, DSM-IV	Self-report	MDD diagnosis, suicidal ideation	Baseline cannabis use increased the odds of meeting criteria for MDD and increased the odds of reporting suicidal ideation at Wave 2.	7
Gage et al., 2015	*N* = 4,561 UK adolescents (aged 16)	Longitudinal (2 year follow-up)	CIS-R	Self-report	MDD diagnosis	After adjusting for other substance use, cannabis use did not predict development of MDD.	7
Halladay et al., 2019	*N* = 43,466 Canadians (ages 15–60)	Repeat Cross-sectional	DSM-IV	Self-report	MDE, past 12-month suicidal ideation	After controlling for other substance use, at least monthly nonmedical cannabis use was associated with an increased odds of a MDE and suicidal ideation.	6
Harder et al., 2006	*N* = 8,759 American adults (ages 29–37)	Longitudinal (1 annual follow-up)	Center for Epidemiologic Studies—Depression (CES-D) questionnaire	Self-report	MDE	After adjusting for covariates, past-year cannabis use did not significantly predict a later MDE.	7
Hassan et al., 2021	*N* = 33,917 American adults (aged 18 and older)	Longitudinal (1 year follow-up)	DSM-5	DSM-5	Occurrence of first MDE	CUD alone did not increase the probability of MDE occurrence after one year. However, comorbidity of CUD and OUD increased the probability of MDE occurrence.	8
Hayatbakhsh et al., 2007	*N* = 3,239 Australian young adults (ages 21)	Longitudinal birth cohort (21 year follow-up)	Young Adult Self Report	Self-report	Depressive symptoms	After controlling for confounding factors, those who started using cannabis before age 15 years and used it frequently at 21 years were more likely to report symptoms of depression in early adulthood	7
Hengartner et al., 2020	*N* = 591 Swiss young adults (ages 19–20)	Longitudinal (30 year follow-up)	DSM-III	Self-report	MDD Diagnosis, suicidality	Cannabis use during adolescence was associated with adult MDD and suicidality. Earlier age of onset and frequent use were associated with a higher risk of MDD in adulthood.	8
Hines et al., 2020	*N* = 1,087 UK young adults (aged 24)	Cross-sectional	DSM-IV	Self-report	MDD diagnosis	No potency of cannabis use was associated with a MDD diagnosis.	8
Leadbeater et al., 2019	*n* = 662 Canadian adolescents (ages 12–18) and *n* = 36,309 US adults (ages 18–64)	Longitudinal adolescent data (10 year follow-up) and cross-sectional adult data	Brief Child and Family Phone Interview, DSM-5	MINI, DSM-5	MDD Diagnoses	Among the adolescent sample, cannabis use was significantly associated with more depressive symptoms from ages 16–19 and following age 25. Associations for depressive symptoms were stronger for males than females at ages 19–20. Among the adult sample, frequent cannabis use was associated with depressive symptoms from ages 18 to 64.	9
Livne et al., 2018	*N* = 36,309 American adults (18 and older)	Cross-sectional	DSM-5	DSM-5	Dysthymia	Logistic regression analyses indicated an elevated likelihood of dysthymia among individuals in both the non-CUD users group and CUD users, compared to non-users.	9
London-Nadeau et al., 2021	*N* = 1,548 Canadian adolescents (ages 13)	Longitudinal (4 year follow-up)	Child Behavior Checklist	Self-report	Depressive symptoms	Cannabis use at 15 years was positively associated with depression symptoms at 17 years for LGB adolescents only when controlling for other substance use.	6
Manrique-Garcia et al., 2012	45,087 Swedish men (ages 18–20)	Longitudinal (35 year follow-up)	ICD Interview	Conscription questionnaire	MDD Diagnosis	After control for confounding factors, there was no association between cannabis use and an increased risk of developing MDD.	7
Marmorstein et al., 2010	*N* = 2,451 American girls (ages 5–8)	Longitudinal (6 annual waves)	DSM-III-R	DSM-III-R	Depressive symptoms	Initial use of cannabis was associated with a specific increase in depressive symptoms among girls who already were experiencing high levels of depressive symptoms at baseline.	7
Marmorstein et al., 2011	*N* = 1,252 American adolescents (ages 17)	Longitudinal (7 year follow-up)	DSM-III-R	DSM-III-R	MDD Diagnosis	Adjusting for adolescent MDD and gender, CUDs in adolescence were associated with an approximately 3-fold increase in risk for later MDD.	7
Medina et al., 2007	*N* = 32 American adolescents (ages 16–18)	Cross-sectional	BDI	Customary Drinking and Drug Use Record (CDDR)	Depressive symptoms	Cannabis use and white matter volume were additive and interactive in predicting depressive symptoms among adolescents.	9
Meier et al., 2020	*N* = 856 American adolescent boys (mean age = 13.4)	Longitudinal (13 year follow-up)	Youth Self Report	Substance Use Questionnaire	Depression problems	After controlling for time-varying covariates, increases in cumulative prior years of weekly cannabis use, but not recent cannabis use, was associated with increases in depression symptoms. There was no evidence of reverse causation.	6
Mustonen et al., 2021	*N* = 6,325 Finnish adolescents (ages 15–16)	Longitudinal (17 year follow-up)	ICD-10	Self-report	MDD Diagnosis	There were significant associations between adolescent cannabis use and later MDD diagnosis after controlling for covariates.	8
Otten et al., 2013	*N* = 310 Dutch adolescents (ages 14–17)	Longitudinal (4 year follow-up)	Depressive Mood List	Self-report	Depressive symptoms	Cannabis use at baseline increased the risk for depressive symptoms, but only in individuals with the short allele of the 5-HTTLPR genotype.	7
Patton et al., 2002	*N* = 1,601 Australian students (ages 14–15)	Longitudinal (7 annual waves)	Clinical interview schedule	Retrospective 7-day diary	MDD Diagnosis	Weekly or more frequent cannabis use in teenagers predicted an approximately twofold increase in risk for later MDD. Daily use in young women was associated with an over fivefold increase in the odds of MDD.	7
Pedersen, 2008	*N* = 2033 Norwegian adolescents (ages 12–16)	Longitudinal (13 year follow-up)	Johns Hopkins Symptom Checklist	Self-report	Depression symptoms, suicide ideation, suicide attempts	Any cannabis use was linked to later suicide ideation. Regular cannabis use was associated with an increased risk of suicide attempt. Any cannabis use was not linked to later depression.	6
Rabiee et al., 2020	*N* = 1,100 Swedish women (ages 18 and over)	Cross-sectional (separate analyses of 3 cohorts)	DSM-III, DSM-IV	Composite International Diagnostic Interview – Substance Abuse Module (CIDI-SAM)	Lifetime diagnosis of MDD	Cannabis use was associated with depression in the youngest cohort of women only.	9
Repetto et al., 2008	*N* = 622 African American youth (ages 15–19)	Longitudinal (6 annual waves)	Brief Symptom Inventory	Self-report	Depressive symptoms	Cannabis use did not predict later depressive symptoms for females or males. Depressive symptoms predicted later cannabis use only for males.	7
Schoeler et al., 2018	*N* = 285 UK male children (aged 8)	Longitudinal (40 year follow-up)	DSM-IV	Self-report	MDD Diagnosis	Early-onset cannabis use (before age 18) but not late-onset cannabis use (after age 27) was associated with a higher risk and shorter time until a subsequent MDD diagnosis. Effect of increased frequency of cannabis use on increased risk of subsequent MDD was observed only for use during adolescence (age 14–18)	7
Shalit et al., 2016	*N* = 77,746 American adults (ages 18 and over)	Longitudinal (3 year follow-up)	DSM-IV	DSM-IV	Suicidal ideation, suicide attempts	Any or daily cannabis use was linked to an increased risk of suicidality in men, but not in women. Conversely, women who reported experiencing suicidality at baseline were more likely to start using cannabis, while no such association was observed in men. There was no association between cannabis use and suicide attempts.	9
van Laar et al., 2007	*N* = 3,881 Dutch adults (ages 18–64)	Longitudinal (3 year follow-up)	DSM-III	Self-report	First MDE	After adjustment for confounders, any use of cannabis at baseline predicted a modest increase in the risk of a first MDE.	8
Wittchen et al., 2007	*N* = 1,395 German adolescents (ages 14–17)	Longitudinal (10 year follow-up)	DSM-IV	DSM-IV	MDD diagnosis	Baseline cannabis use was prospectively associated with a MDD diagnosis.	8
Womack et al., 2016	*N* = 264 low SES American males (ages 11)	Longitudinal (assessments at ages 11, 12, 17, 20, and 22)	Beck Depression Inventory	Self-report	Depressive symptoms	Concurrently, but not longitudinally, cannabis use was positively associated with depressive symptoms.	6

**Table 1B:** Summary of observational studies exploring the relationship between Cannabis use, manic symptoms, and BD diagnoses (12 Studies, *N* = 1,907,623).

Author, year	Study population	Study design	Mood symptom or disorder measure	Cannabis use measure	Outcome	Relevant findings	NOS rating
Agrawal et al., 2011	*N* = 2,232 American adults (ages 16 and over)	Cross-sectional	DSM-IV	DSM-IV	Prevalence of BD	Individuals with BD were significantly more likely to report a lifetime history of cannabis use compared to those without BD.	9
de Hert et al., 2011	*N* = 766 Belgian patients with schizophrenia or BD (ages 16–65)	Cross-sectional	CIDI	CIDI	BD age of onset	Cannabis use was associated with a 9-year reduction in the average age of onset for BD.	9
de Lima Bach et al., 2021	*N* = 1,244 Brazilian young adults (ages 18–24)	Longitudinal (5 year follow-up)	MINI	Alcohol, Smoking and Substance Involvement Screening Test (ASSIST)	New-onset BD	CUD increased the relative risk of BD at follow-up.	8
Denissoff et al., 2022	*N* = 6,325 Finnish adolescents (ages 15–16)	Longitudinal	ICD-10	Self-report	BD Diagnosis	Adolescent cannabis use was not associated with BD after adjusting for other substance and alcohol use.	8
Feingold et al., 2015	*N* = 28,630 US adults (ages 18 and above)	Repeat cross-sectional	DSM-IV	Self-report	BD incidence	Weekly to almost daily use was not significantly associated with increased incidence of BD. Baseline BD was not associated with incidence of cannabis use.	8
Henquet et al., 2006	*N* = 4,815 Dutch adults (ages 18–64)	Longitudinal (3 year follow-up)	CIDI	CIDI	Manic symptoms	Baseline cannabis use increased the risk for manic symptoms during follow-up, even after controlling for confounds. There was no evidence for reverse causality.	9
Lagerberg et al., 2011	*N* = 151 Swedish patients with BD (ages 18–65)	Cross-sectional	DSM-IV	DSM-IV	BD age of onset	Frequent cannabis use was associated with an earlier onset of BD after adjusting for confounds.	7
Lagerberg et al., 2014	*N* = 324 Swedish patients with BD (ages 18–65)	Cross-sectional	DSM-IV	DSM-IV	BD age of onset	There was a significant dose–response relationship between cannabis use and age of onset for first BDE even after adjusting for confounds.	7
Man Xiong Lai et al., 2012	*N* = 1,814,830 Australian patients (ages 10 and older)	Cross-sectional	ICD-10	ICD-10	Prevalence of BD	CUD was significantly associated with the presence of a comorbid BD.	5
Manrique-Garcia et al., 2012	*N* = 45,087 Swedish men (ages 18–20)	Longitudinal (35 year follow-up)	ICD Interview	Conscription questionnaire	BD Diagnosis	After control for confounding factors, there was no association between cannabis use and an increased risk of developing BD.	8
Marwaha et al., 2018	*N* = 3,370 UK adolescents (ages 17)	Longitudinal (6 year follow-up)	Hypomania Checklist Questionnaire	Self-report	Hypomania symptoms	Frequent cannabis use, occurring at least 2–3 times a week, was linked to subsequent hypomania after adjusting for confounds.	7
van Laar et al., 2007	*N* = 3,881 Dutch adults (ages 18–64)	Longitudinal (3 year follow-up)	DSM-III	Self-report	First BDE	After adjustment for confounders, cannabis use at baseline predicted an increase in the risk of a first BDE.	

**Table 2A:** Summary of studies exploring the impact of cannabis on course of illness in MDD (11 Studies, *N* = 4,778).

author, year	Study population	Study design	Mood disorder measure	Cannabis use measure	Outcome	Relevant findings	NOS, RoB, or Robins rating
Bahorik et al., 2017	*N* = 307 American MDD outpatients (mean age = 37)	Prospective (12-month follow-up)	Patient Health Questionnaire	Self-Report	Depression symptoms, anxiety symptoms, mental health functioning	At the 6-month follow-up, individuals using cannabis exhibited significantly less improvement in depression, anxiety, and overall mental health functioning compared to those who did not use cannabis, with no significant difference observed in physical health functioning.	7
Bahorik et al., 2018	*N* = 307 American MDD outpatients (mean age = 37)	Prospective (12-month follow-up)	Patient Health Questionnaire	Self-Report	MDD clinical outcomes	Relative to non-users at baseline, patients using cannabis had worse mental/physical health functioning, higher suicidal ideation, worse mental health functioning, and fewer psychiatry visits. Patients using cannabis over time improved less in depression symptoms and suicidal ideation than non-users.	7
Bovasso, 2001	*N* = 1920 American adults (ages 18 and over)	Longitudinal (2 year follow-up)	DSM-III	DSM-III	Depressive symptoms, MDD diagnosis	Baseline CUD was associated with an increased risk of experiencing depressive symptoms, including suicidal ideation and anhedonia at follow-up. Depressive symptoms at baseline did not predict CUD at follow-up.	9
Cornelius et al., 2010	*N* = 6 American adolescents with MDD and CUD (ages 18–25)	Cross-sectional	DSM-IV	DSM-IV	Amygdala reactivity	Decreases in cannabis use was associated with an increase in threat-related amygdala reactivity levels.	6
Feingold et al., 2017	*N* = 2,348 American adults with MDD (ages 18 and over)	Longitudinal (one year follow-up)	DSM-IV	DSM-IV	Number of depressive symptoms, suicidal ideation, psychosocial functioning, treatment utilization, quality of life	Level of cannabis use was associated with significantly more depressive symptoms at follow-up, particularly anhedonia, changes in body weight, insomnia or hypersomnia and psychomotor problems. After adjusting for baseline confounding factors, no associations were found between cannabis use and suicidality, functionality, and quality of life.	8
Ford et al., 2014	*N* = 75 Canadian adolescents (mean age = 20)	Cross-sectional	DSM-5, Beck Depression Inventory	Timeline Follow Back	Functional brain activation to a rewarding stimulus	The MDD + CU group exhibited heightened activation in prefrontal cortex and striatal regions when exposed to a rewarding stimulus, indicating a unique hyperactivation in response to a non-drug reward, distinct from other study groups.	6
Lucatch et al., 2020	*N* = 14 Canadian adults with MDD and CUD (ages 18–55)	Longitudinal	DSM-5	DSM-5, Urine toxicology	Depressive symptoms, anhedonia, anxiety	Over 28 days, there was a significant reduction in depressive symptoms, a 57.3% reduction in anxiety scores, and a significant reduction in anhedonia (88.3%).	High risk of bias
Nichols et al., 2021	*N* = 74 Canadian young adults (ages 16–23)	Cross-sectional	DSM-IV	DSM-IV	fMRI activation during an emotion regulation task	In comparison to MDD only individuals, individuals with MDD and CU exhibited increased brain activity in response to emotionally positive conditions but reduced activity in response to emotionally negative conditions in the left temporal lobe.	9
Osuch et al., 2016	*N* = 74 Canadian adolescents (ages 16–23)	Cross-sectional	DSM-5	DSM-5	Default mode network connectivity, cognition	Frequent cannabis use or MDD, either separately or in combination, did not result in detectable cognitive impairments. Abnormal brain activity was noted in the fusiform gyrus among participants with both MDD and CU.	9
Secora et al., 2010	*N* = 108 American adults (mean age = 37.5)	Cross-sectional	DSM-IV	DSM-IV	Psychosocial functioning, cognition	CUD + MDD individuals displayed more psychosocial impairments than those with CUD alone. Contrary to the authors’ hypothesis, individuals with CUD + MDD exhibited less impairment in reaction time measurements than those with CUD alone.	7
Sorkhou et al., 2022	*N* = 11 Canadian adults with MDD and CUD (ages 18–55)	Longitudinal	DSM-5	DSM-5, Urine toxicology	Cognition	Visual search speed, selective attention, and visuospatial working memory significantly improved over the 28 days of cannabis abstinence.	High risk of bias

**Table 2B:** Summary of studies exploring the impact of cannabis on course of illness in BD (19 Studies, *N* = 658,278).

Author, year	Study population	Study design	Mood disorder measure	Cannabis use measure	Outcome	Relevant findings	NOS, RoB, or Robins rating
Agrawal et al., 2011	*N* = 2,232 American adults (ages 16 and over)	Cross-sectional	DSM-IV	DSM-IV	Psychosis, Rapid Cycling, Mixed Episodes, General Disability, # of depression episodes, # of mania episodes	Individuals with both BD and CUD experienced greater disability, higher rates of suicide attempts, and an increased likelihood of mixed episodes, even after controlling for confounds.	9
Baethge et al., 2008	*N* = 166 German first-episode BD patients (median age = 28)	Longitudinal (up to 8 year follow-up)	DSM-IV	DSM-IV	Manic and depressive symptoms	Cannabis use significantly predicted severity of manic or hypomanic symptoms in BD but not depressive symptoms.	7
Gruber er al., 2012	*N* = 42 US adults with BD (mean age = 24)	Longitudinal	DSM-IV	DSM-IV	Mood symptoms	Mood changes were observed before and after cannabis use in both the cannabis use + BD groups and cannabis use groups. The CU + BD group showed improved mood scores, while the CU group exhibited an increase in total mood disturbance on the Profile of Mood States.	6
Hartberg et al., 2018	*N* = 591 patients with schizophrenia or BD (mean age = 34)	Cross-sectional	DSM-IV	DSM-IV	Cortical abnormalities, cortical thickness, surface area	There was no significant evidence of compromised brain structure in patients who also used cannabis. However, there was evidence for limited cortical thinning in a group of patients with BD reporting cannabis use prior to illness onset.	5
Kvitland et al., 2016	*N* = 101 BDI Norwegian patients (ages 17–65)	Cross-sectional	DSM-IV	DSM-IV	Mood symptoms	Recent cannabis use had a significant association with the age at onset of the first episodes of mania, psychotic features, and depression symptom severity. Any lifetime suicidal attempt was more prevalent in cannabis users reporting recent use in comparison to non-users with BD.	7
Lagerberg et al., 2016	*N* = 642 French and Norwegian BD patients who reported daily tobacco use (mean age = 35)	Cross-sectional	DSM-IV	DSM-IV	Duration of illness, number of illness episodes, hospitalizations	After controlling for confounds, CUD was associated with earlier BD onset, increased rates of manic episodes, and hospitalizations.	9
Le et al., 2021	*N* = 42 American adolescents (mean age = 21)	Cross-sectional	DSM-5	Daily Drug Taking Questionnaire	Stress-related amygdala activity	Greater amygdala reactivity to acute stress is linked to more frequent cannabis use in BD but not among healthy controls.	7
Lev-Ran et al., 2013	*N* = 1905 American adults with BD (ages 18 years and older)	Cross-sectional	DSM-IV	DSM-IV	BD age of onset, number of BD episodes, treatment utilization, quality of life, mental health disorder comorbidities	Co-occurring CUD is associated with comorbid SUDs, earlier age of onset of first manic and depressive episode, and a greater number of depressive, manic, or hypomanic episodes.	8
Maremmani et al., 2006	*N* = 56 Italian male, psychotic BD patients (mean age = 35; n = 30 with CUD)	Retrospective chart review	DSM-IV	DSM-IV	Hospitalization discharge with clozapine	Patients with CUD had comparable discharge rates from clozapine medication than patients without CU.	5
Oladunjoye et al., 2022	*N* = 266,303 BD hospitalizations in America (mean age = 41)	Retrospective chart review	ICD-9-CM	ICD-9-CM	Medication non-adherence	CUD significantly predicted an increased rate of medication nonadherence among patients with BD even after controlling for potential confounds.	9
Patel et al., 2022	*N* = 380,265 BD American inpatients (mean age = 34.4)	Retrospective chart review	ICD-9	ICD-9	BD-related hospitalization rates	Individuals with Cannabis Use Disorder (CUD) have a significantly increased likelihood of being hospitalized for Bipolar (BP) mania-related concerns but not bipolar-depression-related hospitalizations.	9
Pinto et al., 2023	*N* = 34 Brazilian outpatients with BD (mean age = 44)	Randomized Controlled Trial	DSM-5	150 mg or 300 mg of CBD	BD symptoms, global functioning, remission rates	BD symptoms improved across the full sample, with no significant differences between the CBD and placebo groups. There were no significant differences between the CBD and placebo group on all other clinical rating scales, or in remission rates at endpoint.	Low risk of bias
Strakowski et al., 2007	*N* = 144 American patients with BD (Mean age = 17)	Inception cohort (5-year follow-up)	DSM-IV	DSM-IV	Symptom recovery	Cannabis use was associated with more time in affective episodes and with rapid cycling.	8
Sultan et al., 2021a	*N* = 144 Canadian adolescents (ages 13–20)	Cross-sectional	DSM-IV	DSM-IV	Cortical thickness, surface area (SA), and volume of frontal and parietal regions	Individuals with BD using cannabis showed larger volume and SA in parietal regions but smaller cortical thickness in frontal regions compared to both healthy controls and individuals with BD but no cannabis use.	8
Sultan et al., 2021b	*N* = 134 Canadian adolescents (ages 13–20)	Cross-sectional	DSM-IV	DSM-IV	Functional connectivity	Resting-state functional connectivity between the right orbitofrontal cortex seed and the right lateral occipital cortex was positive in adolescents with BD and lifetime cannabis use, and negative in healthy controls and adolescents with bipolar disorder and no history of cannabis use. Resting-state functional connectivity between the right orbitofrontal cortex seed and right occipital pole was positive in adolescents with BD and lifetime cannabis use, and negative in adolescents with BD and no history of cannabis use.	8
Sultan et al., 2023	*N* = 121 Canadian adolescents (ages 13–20)	Cross-sectional	DSM-IV	DSM-IV	Working memory, attentional set-shifting, inhibitory control, visuospatial working memory, decision-making	Individuals BD and a history of lifetime cannabis use showed lower performance in visuospatial working memory compared to healthy controls. However, there was no significant difference in visuospatial working memory between individuals with BD and a history of lifetime cannabis use and those with BD but no history of cannabis use.	8
van Rossum et al., 2009	*N* = 3,459 European in- and outpatients with BD (mean age = 44.6)	Longitudinal (1 year follow-up)	Clinical Global Impressions Scale	Self-report	BD symptoms, psychosocial functioning, treatment compliance	Over a 12-month period of treatment, patients who used cannabis showed lower levels of compliance and higher overall illness severity, including increased rates of mania and psychosis when compared to non-users. Furthermore, cannabis users reported lower satisfaction with their quality of life than non-users.	8
Weinstock et al., 2016	*N* = 230 adult American inpatients with BD-I (mean age = 42)	Retrospective chart review	DSM-IV-TR	DSM-IV-TR	Comorbid mental health disorder, age of BD onset, clinical characteristics of BD	CUD comorbidity was significantly associated with younger age, manic/mixed episode polarity, presence of psychotic features, and comorbid nicotine dependence, alcohol use disorder (AUD), and other substance use disorders, but was associated with a decreased likelihood of anxiety disorder comorbidity	8
Zorrilla et al., 2014	*N* = 1922 European patients with BD (mean age = 45)	Longitudinal (2 year follow-up)	DSM-IV, ICD-10	Self-report	Functional and clinical outcomes	At follow-up, previous users had similar outcomes to never users, while current users exhibited lower recovery and remission, higher recurrence, increased work impairment, and were more likely to be living without a partner compared to never users.	5

The complete risk of bias assessment for each study is reported in Tables 3, 4. Concerning the NOS ratings, 78.7% (59 out of 75) of the studies exhibited high methodological quality, with the remaining studies indicating moderate methodological quality (21.3%; comprising 16 out of 75 studies). The ROBINS-I tool was applied to two studies conducted by the same research group (27, 28), where both studies displayed a high risk of bias. The only randomized controlled trial included in the review demonstrated a low risk of bias.

**Table 3:** Newcastle Ottawa Scale (NOS) ratings for observational studies.

Author, year (Ref #)	Selection				Comparability		Outcome or exposure			Total points
	1	2	3	4	1a*	1b	1**	2	3	
Agrawal et al., 2011 (29)	✓	✓	✓	✓	✓	✓	✓	✓	✓	9
Agrawal et al., 2017 (30)	✓	✓		✓	✓	✓	✓	✓	✓	8
Baethge et al., 2008 (31)	✓	✓	✓	✓			✓	✓	✓	7
Bahorik et al., 2017 (32)	✓	✓		✓	✓	✓		✓	✓	7
Bahorik et al., 2018 (33)	✓	✓		✓	✓	✓		✓	✓	7
Bataineh et al., 2023 (34)	✓	✓		✓		✓		✓	✓	6
Bolanis et al., 2020 (35)	✓	✓	✓	✓	✓	✓		✓	✓	8
Bovasso, 2001 (36)	✓	✓	✓	✓	✓	✓	✓	✓	✓	9
Carra et al., 2019 (37)	✓	✓	✓	✓		✓		✓	✓	7
Chabrol et al., 2021 (38)	✓	✓		✓	✓	✓		✓	✓	7
Crane et al., 2015 (39)	✓	✓		✓	✓	✓		✓	✓	7
Cornelius et al., 2010 (40)	✓	✓	✓				✓	✓	✓	6
Danielsson et al., 2016 (41)	✓	✓			✓	✓		✓	✓	6
Davis et al., 2023 (42)	✓	✓		✓	✓	✓		✓	✓	7
De Hert et al., 2011 (43)	✓	✓	✓	✓	✓	✓	✓	✓	✓	9
De Lima Bach et al., 2021 (44)	✓		✓	✓	✓	✓	✓	✓	✓	8
Denissoff et al., 2022 (45)	✓	✓		✓	✓	✓	✓	✓	✓	8
Durdle et al., 2008 (46)	✓	✓		✓			✓	✓	✓	6
Espada et al., 2011 (47)	✓	✓		✓	✓	✓		✓	✓	7
Fairman et al., 2012 (48)	✓	✓	✓	✓	✓	✓		✓	✓	8
Feingold et al., 2015 (49)	✓	✓	✓		✓	✓	✓	✓	✓	8
Feingold et al., 2017 (19)	✓	✓	✓		✓	✓	✓	✓	✓	8
Fleming et al., 2008 (50)	✓	✓			✓	✓		✓	✓	6
Ford et al., 2014 (51)	✓	✓	✓	✓				✓	✓	6
Frohe et al., 2019 (52)	✓	✓			✓	✓	✓	✓	✓	7
Gage et al., 2014 (53)	✓	✓		✓	✓	✓	✓		✓	7
Gruber et al., 2012 (54)	✓	✓	✓	✓				✓	✓	6
Halladay et al., 2019 (55)	✓	✓			✓	✓		✓	✓	6
Harder et al., 2006 (56)	✓	✓			✓	✓	✓	✓	✓	7
Hartberg et al., 2018 (57)	✓	✓	✓					✓	✓	5
Hassan et al., 2021 (58)	✓	✓	✓		✓	✓	✓	✓	✓	8
Hayatbakhsh et al., 2007 (59)	✓	✓		✓	✓	✓		✓	✓	7
Hengartner et al., 2020 (60)	✓	✓		✓	✓	✓	✓	✓	✓	8
Henquet et al., 2006 (21)	✓	✓	✓	✓	✓	✓	✓	✓	✓	9
Hines et al., 2020 (61)	✓	✓	✓	✓	✓	✓		✓	✓	8
Kvitland et al., 2016 (62)	✓	✓	✓	✓			✓	✓	✓	7
Lagerberg et al., 2011 (63)	✓	✓			✓	✓	✓	✓	✓	7
Lagerberg et al., 2014 (64)	✓	✓			✓	✓	✓	✓	✓	7
Lagerberg et al., 2016 (65)	✓	✓	✓	✓	✓	✓	✓	✓	✓	9
Leadbeater et al., 2019 (66)	✓	✓	✓	✓	✓	✓	✓	✓	✓	9
Le et al., 2021 (67)	✓	✓	✓			✓	✓	✓	✓	7
Lev-Ran et al., 2013 (68)	✓	✓	✓	✓		✓	✓	✓	✓	8
Livne et al., 2018 (69)	✓	✓	✓	✓	✓	✓	✓	✓	✓	9
London-Nadeau et al., 2021 (70)	✓	✓			✓	✓		✓	✓	6
Manrique-Garcia et al., 2012 (71)	✓	✓		✓	✓	✓	✓	✓	✓	7
Man Xiong Lai et al., 2012 (72)	✓	✓					✓	✓	✓	5
Marmorstein et al., 2009 (73)	✓	✓	✓	✓			✓	✓	✓	7
Marmorstein et al., 2011 (74)	✓	✓	✓	✓			✓	✓	✓	7
Maremmani et al., 2006 (75)	✓			✓			✓	✓	✓	5
Marwaha et al., 2018 (76)	✓	✓		✓	✓	✓		✓	✓	7
Medina et al., 2007 (77)	✓	✓	✓	✓	✓	✓	✓	✓	✓	9
Meier et al., 2020 (20)	✓	✓			✓	✓		✓	✓	6
Mustonen et al., 2021 (78)	✓	✓		✓	✓	✓	✓	✓	✓	8
Nichols et al., 2021 (79)	✓	✓	✓	✓	✓	✓	✓	✓	✓	9
Oladunjoye et al., 2022 (80)	✓	✓	✓	✓	✓	✓	✓	✓	✓	9
Osuch et al., 2016 (81)	✓	✓	✓	✓	✓	✓	✓	✓	✓	9
Otten et al., 2013 (82)	✓	✓		✓	✓	✓		✓	✓	7
Patel et al., 2022 (83)	✓	✓	✓	✓	✓	✓	✓	✓	✓	9
Patton et al., 2002 (84)	✓	✓			✓	✓	✓	✓	✓	7
Pedersen, 2008 (85)	✓	✓		✓		✓		✓	✓	6
Rabiee et al., 2020 (86)	✓	✓	✓	✓	✓	✓	✓	✓	✓	9
Repetto et al., 2008 (87)	✓	✓		✓	✓	✓		✓	✓	7
Secora et al., 2010 (88)	✓	✓		✓		✓	✓	✓	✓	7
Schoeler et al., 2018 (89)		✓		✓	✓	✓	✓	✓	✓	7
Shalit et al., 2016 (90)	✓	✓	✓	✓	✓	✓	✓	✓	✓	9
Strakowski et al., 2007 (91)	✓	✓		✓	✓	✓	✓	✓	✓	8
Sultan et al., 2021a (92)	✓	✓	✓	✓		✓	✓	✓	✓	8
Sultan et al., 2021b (93)	✓	✓	✓	✓		✓	✓	✓	✓	8
Sultan et al., 2023 (94)	✓	✓	✓	✓		✓	✓	✓	✓	8
van Laar et al., 2007 (95)	✓	✓		✓	✓	✓	✓	✓	✓	8
van Rossum et al., 2009 (96)	✓		✓	✓	✓	✓	✓	✓	✓	8
Weinstock et al., 2016 (97)	✓		✓	✓	✓	✓	✓	✓	✓	8
Wittchen et al., 2007 (98)	✓	✓	✓	✓		✓	✓	✓	✓	8
Womack et al., 2016 (99)	✓	✓			✓	✓		✓	✓	6
Zorrilla et al., 2015 (100)	✓	✓		✓				✓	✓	5

**Table 4:** Risk of bias using Cochrane’s risk of bias in non-randomized studies of interventions tool (ROBINS-I) and Cochrane’s risk of bias tool (RoB-2).

Author, year	Randomization	Allocation concealment	Blinding of participants and personnel	Blinding of outcome assessment	Incomplete outcome data	Selective reporting	Other bias	Overall risk of bias
Lucatch et al., 2020 (27)	High	Low	Some concerns	Low	Low	Low	Low	High
Pinto et al., 2023 (26)	Low	Low	Low	Low	Low	Low	Low	Low
Sorkhou et al., 2022 (28)	High	Low	Some concerns	Low	Low	Low	Low	High

### Relationship between cannabis use, depressive symptoms and MDD diagnoses

Nine studies examined the cross-sectional relationship between cannabis use and depressive symptoms or MDD diagnoses, with seven identifying significant associations (30, 46–48, 69, 86, 101). In a retrospective analysis of data from the Australian Twin Registry, frequent cannabis use (≥ 100 times across their lifetime) was associated with MDD and suicidal ideation in both monozygotic and dizygotic twins (30). In a similar study using nationally representative data to investigate the relationship between cannabis use and a past-year experience of a major depressive episode (MDE), Fairman et al. (48) found that, compared to individuals with no cannabis use, those reporting both early-onset and adult-onset cannabis use had an increased likelihood of experiencing a MDE, even after adjusting for other substance use (with odds ratios of 1.7 and 1.8, respectively). These findings contrast with an epidemiological study conducted in the UK among young adults, where self-reported use of high- or low-potency cannabis did not correlate with a diagnosis of MDD after controlling for relevant factors, including other substance use (61). Similarly, Chabrol et al. found no significant association between cannabis use disorder (CUD) with suicidal ideation or depressive symptoms in a sample of 1,034 French postsecondary students (38).

In two smaller cross-sectional studies that examined the role of cannabis use in depressive symptoms among youth, cannabis use emerged as a significant predictor of this outcome (47, 101). Medina and colleagues (101) demonstrated that adolescent cannabis users, in comparison to never users, had exhibited neural abnormalities, including reduced hippocampal volumes and white matter alterations, along with higher levels of depressive symptoms. In a separate study examining depressive symptoms among 707 Spanish youth, Espada and colleagues (47) found that, after controlling for alcohol and tobacco use, adolescents who reported past 30-day cannabis use reported significantly higher levels of depressive symptoms than non-users.

Thirty-one studies prospectively explored the relationship between cannabis use and subsequent depression, including depressive symptoms, MDD diagnoses, and suicidality. Among these studies, 22 identified a significant temporal association between baseline cannabis use and subsequent depression. Cannabis use was a significant predictor of adulthood depression across 12 sample sets following adolescents (20, 34, 42, 59, 60, 66, 70, 74, 78, 82, 84, 89). In a sample of LGB and heterosexual adolescents, London-Nadeau and colleagues observed that cannabis use at age 13 predicted depression symptoms at ages 15 and 17 among LGB participants only (70). However, a bidirectional relationship was also identified among LGB adolescents, where baseline depressive scores predicted subsequent cannabis use. In one study that tracked a cohort of Australian young adults from birth, and after controlling for covariates, individuals who used cannabis at least once a week were more likely to report depressive symptoms than those who never used cannabis or those who used cannabis less than weekly (42). Furthermore, among young adults who used cannabis at least weekly, the risk of reporting depressive symptoms was greater among those who initiated cannabis use before the age of 15. Additionally, one study prospectively explored the relationship between cannabis use, depressive symptoms, and the serotonin transporter gene (5-HTTLPR) in adolescents (82). The serotonin transporter gene is highly regarded as a key candidate gene due to its involvement in development of depression and other mental health disorders [see (102)]. Specifically, this gene encodes the serotonin transporter protein, which plays a crucial role in reuptake of serotonin into presynaptic neurons. The authors found that cannabis use elevated the risk of experiencing heightened depressive symptoms over a 5-year period, but only among people with the short allele of the 5-HTTLPR genotype.

In prospective studies that identified gender-specific effects between cannabis use and subsequent depression, findings were mixed. In a nationally representative study of American youth and adults by Carra and colleagues (37), it was found that women who reported cannabis use at baseline were more likely than men to experience a major depressive episode (MDE) 1 year later. Moreover, among adults only, higher potency cannabis use predicted a subsequent MDE. Similarly, in a study that tracked cannabis use trends among Australian adolescents over a seven-year period, daily cannabis use was associated with a more than five-fold increase in depressive symptoms among girls, but not among boys (84). In contrast, three studies detected stronger associations between cannabis use and subsequent depressive symptoms in men (39, 50, 66, 90). A longitudinal study spanning 4 years and involving 951 adolescents observed that increases in cannabis use were associated with increases in the severity of depressive symptoms in boys only (50).

Three prospective studies examined the link between cannabis use and suicidality, with all reporting significant associations (55, 85, 90). In a study that followed adolescents for 13 years, the authors found that while frequent or any cannabis use did not predict later depression, any cannabis use was linked to later suicidal ideation, and frequent use was associated with suicide attempts (85).

However, nine longitudinal studies did not establish a temporal relationship between cannabis use and subsequent depressive symptoms or diagnoses when controlling for relevant confounding variables, including other alcohol or substance use (35, 41, 49, 56, 58, 71, 87, 99, 103). In a recent study examining the relationship between various substance use disorders (SUDs) and the incidence of a first MDE, Hassan et al. (58) found that CUD alone did not increase the likelihood of experiencing a MDE 1 year later. However, the comorbidity of CUD and opioid use disorder (OUD) significantly increased this association. Moreover, two prospective studies found that while baseline cannabis use did not predict a diagnosis of MDD at follow-up, baseline severity of depressive symptoms was predictive of subsequent cannabis use (49, 87).

### Relationship between cannabis use, mania, and BD diagnoses

Five studies examined the cross-sectional association between cannabis use and the presence of (hypo) manic symptoms or the diagnosis of BD, all of which identified a significant relationship (29, 43, 63, 64, 72). Lagerberg and colleagues (64) found a significant dose-dependent relationship between cannabis use and age of onset for BD. This effect of cannabis was not confounded by multiple variables, including gender, family history of BD, lifetime psychotic symptoms, and other substance or alcohol use. Similarly, in a retrospective chart review of more than one million Australian patients, which focused on the relationship between SUDs and mental health disorders, Lai and colleagues found a significant association between CUD and BD diagnoses (72).

Moreover, seven studies prospectively examined the relationship between cannabis use and BD diagnoses and symptoms, with four establishing a significant association (21, 44, 76, 95). In a nationally representative sample of Dutch adults, cannabis use at baseline nearly tripled the risk of reporting manic symptoms at a 3-year follow-up, even after controlling for confounds (21). No evidence of reverse causality was found. Similarly, Marwaha et al. (76) found that among 3,370 UK adolescents, cannabis use at least 2–3 times per week at baseline doubled the risk of reporting manic symptoms at a 6-year follow-up.

However, three studies did not detect a temporal relationship between cannabis use and BD diagnoses or symptoms (45, 49, 71). Using Waves 1 and 2 of the National Epidemiologic Survey on Alcohol and Related Conditions (NESARC), Feingold et al. (49) found that baseline cannabis use was not associated with an increased incidence of BD or MDD when controlling for other substance use. In a separate epidemiological study that examined lifetime cannabis exposure and its association with BD within a Finnish birth cohort, no association was found between adolescent cannabis use and BD diagnosis in adulthood after adjusting for alcohol use, smoking, and lifetime drug use (45).

### Impact of cannabis on the course of MDD

Five cross-sectional studies evaluated the effects of cannabis use in patients with MDD (40, 51, 79, 81, 88), with three identifying neural abnormalities compared to controls (40, 51, 79). Ford et al. (51) employed a passive music listening paradigm to investigate variations in reward processing in brain activity across four groups of youth: healthy controls, frequent cannabis users, individuals with MDD, and individuals with MDD who use cannabis. They observed significant hyperactivation in prefrontal and limbic regions during the task only within the MDD cannabis-using group. In a study assessing cognition and psychosocial functioning, Secora and colleagues found that adults with comorbid CUD and MDD reported more psychosocial impairments than those with CUD alone (88). However, when assessing memory, verbal skills, and attention, there were no significant differences between the two groups. Similar findings were noted in a more recent study that examined cognitive functioning and neural activity across four groups of adolescents: healthy controls, youth with current MDD without frequent cannabis use, youth with frequent cannabis use, and youth with frequent cannabis use and MDD (81). The authors found no evidence of cognitive impairments in any of the cannabis-using groups when compared to non-users. Furthermore, there was no interaction observed between MDD and cannabis use on resting state fMRI connectivity.

When considering the longitudinal effects in MDD, six prospective studies demonstrated an adverse effect of cannabis use (19, 27, 28, 32, 33, 36). In a recent open-label, single-arm investigation utilizing monetary contingency management (CM), the cognitive and clinical effects of 28 days of cannabis abstinence were examined in people with co-occurring MDD and CUD (27, 28). With 8/11 participants achieving cannabis abstinence as confirmed by urine toxicology, significant improvements were observed in depressive symptoms and anhedonia (27), and in specific cognitive areas including visual search speed, visual sustained attention, response inhibition, and visuospatial working memory (28). Moreover, in a secondary analysis of a randomized controlled trial involving 307 outpatients with MDD receiving substance/alcohol use treatment, patients using cannabis for medical purposes experienced worse mental and physical health functioning at study endpoint, in comparison to non-users (32, 33). Furthermore, non-medical use of cannabis was linked to higher suicidal ideation, poorer global functioning, and fewer psychiatry visits. Over time, patients using non-medical cannabis exhibited less improvement in depression symptoms and suicidal ideation compared to non-users. Utilizing data from Waves 1 and 2 of NESARC, Feingold et al. (19) explored the effect of cannabis use and CUD on the course and outcome of MDD over a three-year period. Cannabis use at baseline was associated with a significant increase in depressive symptoms at follow-up, but did not correlate with suicidality, quality of life, or psychosocial functioning. Similar findings were obtained by Bovasso (36) where baseline CUD was predictive of worsened depressive symptoms, suicidal ideation, and anhedonia in a community sample of adults with MDD over a 2-year period.

### Impact of cannabis on the course of BD

Of 13 cross-sectional studies that investigated cannabis use in BD, 11 identified a negative impact associated with this substance (29, 57, 62, 64, 67, 68, 80, 83, 92, 93, 97). In one study examining clinical characteristics associated with comorbid CUDs in BD inpatients, Weinstock et al. (97) found that CUD comorbidity was significantly associated with a greater presence of psychotic features, diagnosis at a younger age and a greater likelihood of meeting diagnostic criteria for an alcohol use disorder (AUD) or other SUD. Other evidence indicates that cannabis use in BD is associated with greater severity of depressive and manic symptoms (29, 62, 64, 68), an increased number of hospitalizations (64, 83), reduced medication adherence (80), greater disability (29) and a higher number of suicide attempts (29, 62). Moreover, two studies found that compared to BD-only patients, those with comorbid CUD exhibited a reduction in cortical thickness in frontal brain regions (57, 93). Similarly, Sultan and colleagues (92) observed that patients with BD and comorbid CUD demonstrated reduced resting-state functional connectivity across multiple frontal-temporal regions, in contrast to those with BD only.

However, two studies did not find a significant relationship between cannabis use and BD-related outcomes (75, 94). In a retrospective chart review, Maremmani and colleagues discovered that, contrary to their initial hypothesis, hospital discharge rates were comparable between BD patients with CUD and BD patients without cannabis use (75). In a separate study that compared various cognitive outcomes among three distinct groups, including adolescents with BD and no history of cannabis use, those with BD and cannabis use, and healthy controls without cannabis use, the authors found that the BD and cannabis use group and the BD group exhibited similar cognitive performance, which was inferior to controls (94). This finding contradicted the researchers’ initial hypothesis that cannabis use exacerbates cognitive impairments in adolescents with BD.

Five of the studies included in the review prospectively explored the effects of cannabis in BD. All of these studies (31, 91, 96, 100), except one (104), revealed negative outcomes linked to cannabis use. In a 2-year prospective observational study, individuals with BD who continued using cannabis demonstrated lower recovery rates and remission, in addition to poorer psychosocial functioning than never users and those who reduced their cannabis use (100). In a study examining clinical outcomes among a sample of 3,459 European patients diagnosed with BD, those who self-reported cannabis use exhibited reduced treatment compliance, increased illness severity, and lower satisfaction with their quality of life at a one-year follow-up (96). Other evidence suggests that cannabis use in BD is temporally associated with severity of mania (31), greater number of manic and depressive episodes (91), and rapid cycling (91).

However, in a prospective observational study that examined the immediate effects of smoking cannabis on mood in BD, results showed that the BD group experienced significant mood improvement compared to the group who used cannabis without BD (54). This improvement was evident in reductions in total mood disturbance as measured by the Profile of Mood States. These findings provide empirical support for anecdotal reports indicating that cannabis is sometimes used to alleviate mood-related symptoms (e.g., alleviation of cannabis withdrawal) in at least a subset of people with BD.

In the only randomized controlled trial (RCT) included in our review, Pinto and colleagues (26) investigated the potential of adjunctive cannabidiol (CBD; 0, 150 and 300 mg/day) for BD treatment. BD. Both the CBD and placebo groups exhibited a significant reduction in BD symptoms at study endpoint (Week 8), with no significant between group differences. Adverse events were comparable between groups, indicating that CBD could be a safe and well-tolerated treatment option.

## Discussion

This systematic review provides a comprehensive assessment of the impact of cannabis use on the development of mood disorders and associated symptoms, specifically emphasizing MDD and BD. Furthermore, we examined how cannabis use impacts prognosis and treatment outcomes in people with MDD or BD. Our findings suggest that cannabis use is associated with elevated depressive and manic symptoms in the general population, as well as an increased risk of developing MDD and BD. Furthermore, there is minimal evidence to support the notion that cannabis improves clinical or treatment outcomes for MDD or BD; instead, its use is associated with a poorer prognosis for both disorders (see Table 5).

**Table 5:** Overall trends in cannabis use and mood disorders.

Clinical domain	Number of studies supporting an adverse effect of cannabis	Number of studies supporting no effect of cannabis	Number of studies supporting a therapeutic effect of cannabis	General findings
MDD Symptom or Diagnosis Development	29	11	0	Cannabis use may be moderately associated with elevated depressive symptoms or increased risk of developing MDD (72.5% of studies).
BD Symptom or Diagnosis Development	9	3	0	Cannabis use may be moderately associated with elevated manic symptoms or increased risk of developing BD (75% of studies)
Prognosis of MDD	10	1	0	Cannabis use does not appear to improve MDD-related outcomes and is associated with poorer treatment outcomes in MDD (90.9% of studies)
Prognosis of BD	15	3	1	Cannabis use is associated with poorer treatment outcomes in BD (78.9% of studies).

Our review serves as an extension of prior reviews and meta-analyses by identifying the associations between cannabis use and depressive and manic symptoms across both clinical and non-clinical populations. While previous reviews have focused particularly on specified clinical populations (105), or solely examined the prospective risk associated with cannabis use and mood disorders (106), our review uniquely bridges these perspectives. Furthermore, our review conducts a thorough investigation into the multifaceted impact of cannabis on a range of outcomes within MDD and BD. This exploration extends beyond the traditional focus on mood symptoms, encompassing critical domains such as cognition and neural functioning. We also examine the potential therapeutic effects of cannabinoids in mood disorders, noting that only one trial met the specified criteria (26). Overall, this expanded analysis enriches our comprehension of the intricate relationship between cannabis use and mood-related outcomes, offering a more holistic and comprehensive perspective.

### Cannabis use, mood symptoms, and mood disorder diagnoses

We found that cannabis use is associated with elevated depressive symptoms, mania, and suicidality. Consistent with other reviews and meta-analyses (107–109), a dose-dependent relationship has been observed between the duration of cannabis use, particularly when initiated in adolescence, and the heightened occurrence of symptoms related to depression and mania [e.g., (37, 64)]. These findings underscore the critical role of age of onset in cannabis use. Adolescence is a period of dynamic brain development, marked by significant structural and functional changes, including maturation of key brain regions implicated in mood regulation and emotional processing (110, 111). This period represents a heightened vulnerability to the potential long-term consequences of environmental stressors, including THC exposure. Indeed, a recent review of over 30 human magnetic resonance imaging studies comparing former cannabis users with control groups has highlighted the emergence of neuroanatomical abnormalities among cannabis users in regions with a high density of CB1 receptors (112).

However, our review obtained some evidence that the relationship between cannabis and mood disorders may be influenced by additional factors, such as concurrent substance use (41, 45, 49). People who engage in cannabis use often co-use other substances (113, 114). Importantly, these co-occurring substances also have the potential to impact brain development and influence mood. This underscores the critical need to account for other substance use when investigating the complex relationship between cannabis use and mood disorders.

### Cannabis and the course of mood disorders

In MDD, we found compelling evidence indicating that cannabis use and CUD are linked to more pronounced symptomatology and a less favorable prognosis compared to patients who do not use cannabis. This includes greater levels of anhedonia (27, 36), suicidality (33), reduced adherence (33) and poorer cognition (28). Notably, a study assessing the effects of 28 days of cannabis abstinence demonstrated substantial enhancements in specific cognitive domains and depressive symptoms (27, 28). These findings imply that the adverse effects of cannabis use may be reversible. Nonetheless, it is imperative to acknowledge a significant limitation in this study—the absence of a non-abstinent control group. This limitation underscores the need for further controlled, long-term research to elucidate the impact of cannabis on clinical outcomes and cognition in MDD. Interestingly, other data suggests that cannabis use in MDD have better cognitive functioning than those who use cannabis without MDD (88). These findings underscore the need for more controlled research on cannabis and cognition in MDD.

Our systematic review also revealed a multitude of adverse consequences of cannabis use in people with BD. This includes more severe symptom profiles (29, 31, 96), a greater number of manic and depressive episodes (62, 64), more rapid cycling (91) and higher levels of suicidality (29). Intriguingly, one study suggested that people with BD who use cannabis experienced greater immediate mood improvements than cannabis-using controls (104). This observation is in line with other research indicating that people with psychiatric disorders may use cannabis for short-term symptom relief (e.g., withdrawal alleviation) (115, 116), despite adverse long-term consequences.

Moreover, we found evidence that baseline manic or depressive symptoms were predictive of subsequent cannabis initiation [e.g., (37, 50)]. These findings have significant implications for treatment, suggesting that following the onset of a mood disorder, people may be more prone to initiate cannabis use. Given the available evidence indicating that cannabis use may be detrimental to people with MDD and BD, this association warrants further study. Thus, it is crucial for clinicians and researchers to explore alternative, evidence-based therapeutic approaches for people with mood disorders who may use cannabis for symptom management.

We found no compelling evidence supporting the therapeutic potential of cannabinoids in mood disorders. The only RCT included in our review that examined CBD’s efficacy and safety for BD yielded inconclusive clinical results. Nonetheless, the findings indicate CBD’s potential as a safe option, and further extensive clinical trials are warranted. Other research has evaluated cannabinoids such as dronabinol, nabilone, and nabiximols on depressive symptoms (117, 118). These studies have been conducted within the context of treatment studies focused on other primary conditions, notably, chronic pain disorders. While there is some evidence that cannabinoids may offer benefits for mood and anxiety symptoms in specific patient populations (119), it is important to note that these people may not necessarily meet the clinical criteria for mood disorders. Overall, there is a need for further investigation, particularly in clinical cohorts with established mental health diagnoses.

### Strengths and limitations

This systematic review has several notable strengths. First, the review encompassed a comprehensive range of studies, including both cross-sectional and prospective designs. This ensured a thorough examination of relationships between cannabis use and mood disorders, providing a holistic view of available evidence. Furthermore, we investigated a diverse set of outcomes. By considering various dimensions, such as depressive and manic symptoms, diagnostic outcomes, cognition, neuroimaging and treatment outcomes, this review provides multifaceted assessment of the impact of cannabis use on mood disorders.

However, there were several limitations. The inherent heterogeneity across the included studies, including variations in methodology, outcome measures, and participant characteristics is a significant limitation. For example, measurements of cannabis use exhibited substantial variability across studies, with many relying on single-item self-reports and few employing objective measures. This lack of standardized assessments of cannabis use introduces variability that may impact our findings. Future research would benefit from more comprehensive methods for quantifying cannabis use (e.g., urine toxicology). Second, few studies examined variables associated with cannabis use, including potency and route of administration, in relation to our outcomes. Two studies (36, 50) examined the relationship between low and high potency cannabis use and depressive symptoms and diagnoses, yielding mixed findings. In contrast, only one study (68) delved into the association between cannabis potency and the initial onset of a bipolar disorder episode, revealing a positive relationship. Relatedly, while only one of the included studies captured data on the route of cannabis administration, no analyses were reported on its relationship with mood symptoms (98). The route of cannabis administration may influence mood-related symptoms through its impact on the speed of onset, bioavailability, peak concentrations, metabolism, and the context of use (120–122). Thus, studying these factors may be crucial for developing a nuanced understanding of how cannabis use may affect mental health. Furthermore, the review predominantly relies on observational studies, which inherently limited our ability to establish causality or determine the direction of the relationship between cannabis use and mood disorders. Moreover, several studies did not control for important confounding factors such as other substance misuse or psychosocial variables. Finally, the majority of included studies did not conduct gender-specific analyses, despite existing evidence indicating gender differences in the effects of cannabis use in the general population (123, 124). This adds to the heterogeneity across studies and calls for more gender-inclusive research to better understand the nuances of this relationship.

## Conclusion

We found that cannabis use is linked to increased depressive and manic symptoms in the general population, and an elevated risk of developing MDD and BD. Moreover, there was evidence of cannabis-related harms in mood disorders. While these findings should be interpreted with caution when considering the observational nature of most eligible studies, the potential implications of these findings are substantial for practitioners and policymakers. Mental health practitioners should incorporate cannabis use screening into their standard assessments, ensuring that people with mood disorders are aware of the potential risks associated with cannabis use. Patients with mood disorders may use cannabis to manage mood symptoms, and clinicians should consider evidence-based alternatives for symptom management. Policymakers and public health officials may consider the need for targeted interventions and education programs focusing on the risks of cannabis use, particularly among people at risk for, or diagnosed with mood disorders.

Finally, a deeper exploration of the neurobiological underpinnings and causal relationships between cannabis use and mood disorders, both in human studies and animal models, would provide a more comprehensive understanding of the mechanisms involved. Moreover, well-controlled prospective studies with comprehensive assessments of cannabis use are needed to further elucidate the nuances of this relationship, while also considering sex- and gender-specific analyses. These studies will advance our knowledge and ultimately improve the mental health and well-being of people impacted by mood disorders.

## Data availability statement

The data analyzed in this study is subject to the following licenses/restrictions: the data that support the findings of this study are available on request from the corresponding author. Requests to access these datasets should be directed to tony.george@camh.ca.

## Author contributions

MS: Data curation, Methodology, Writing – original draft, Writing – review & editing. ED: Data curation, Writing – review & editing. TG: Conceptualization, Funding acquisition, Supervision, Validation, Writing – review & editing, Resources, Writing – original draft.
